# Unique Anatomical Variations of the Lateral Femoral Cutaneous Nerve

**DOI:** 10.7759/cureus.89354

**Published:** 2025-08-04

**Authors:** Milena Douglas, Matthew Mckoy, Jonathan Rozeboom, Alexzander Rich, Ainsley Durning, Dominik Valdez, Heather F Smith, Leigha M Lynch

**Affiliations:** 1 Osteopathic Medicine, Arizona College of Osteopathic Medicine (AZCOM) Midwestern University, Glendale, USA; 2 Anatomy, Arizona College of Osteopathic Medicine (AZCOM) Midwestern University, Glendale, USA

**Keywords:** anatomic variation, dissection, human donor, inguinal, lumbosacral plexus

## Abstract

This case study focuses on the atypical nerve contributions and branching patterns of the lumbar plexus in two human body donors at Midwestern University. It discusses their implications for pathology and surgical outcomes. Variations were identified in the anterior rami contributions and branching patterns of the lumbar plexus in both donors, predominantly in the lateral femoral cutaneous nerve (LFCN). While the typical contributions of LFCN include L2 and L3 spinal cord levels, the case donors received contributions only from L2 or from L1-L3 levels, with the nerves combining and separating multiple times before ultimately forming LFCN or multiple branches thereof. Unique branching patterns were also noted for the ilioinguinal and femoral nerves. These findings suggest that a deeper understanding of the lumbar plexus's variability is necessary to reduce the risks associated with surgical procedures in these regions, such as hip replacement, nerve block, and inguinal hernia repair, as well as the recognition and treatment of atypical nerve branch impingement. Further studies of lumbar plexus nerve contributions will be beneficial for improved clinical knowledge and reducing risks for complications.

## Introduction

The lumbar plexus is a complex structure in the human body, responsible for supplying both motor and sensory innervation to the lower limbs, pelvis, and lower abdominal wall. It consists of a complex network of nerves formed by the anterior rami of the lumbar spinal nerves, arising from L1-L4 vertebral levels, with some individuals also receiving contributions from T12 [1-4]. Understanding the intricate organization of this anatomical structure is crucial as it pertains to nerve block administration and non-pharmacological management of chronic pain, as well as diagnostic and surgical interventions. Anatomical variation among individuals can present a challenge to exercising safe and effective health practices.

Anatomy of the lumbar plexus

The typical anatomical organization of the lumbar plexus, as understood through common anatomical texts, is described hereafter following the listed references [5-7]. The iliohypogastric nerve derives from the anterior rami of T12 and L1 and is the most superiorly positioned nerve within the lumbar plexus. It typically courses on the anterior surface of the quadratus lumborum. This nerve provides innervation to the transversus abdominis and internal abdominal oblique muscles. After providing this motor innervation, the iliohypogastric nerve splits into cutaneous sensory branches that reach the upper lateral gluteal region and a small area of skin superior to the pubis. The ilioinguinal nerve (L1) can commonly be seen arising from the same trunk as the iliohypogastric nerve and traversing inferior to it. The ilioinguinal nerve then passes inferiorly through the inguinal canal and exits the superficial inguinal ring. This nerve supplies motor innervation to the inferior portions of the transversus abdominis and internal abdominal oblique muscles. Additionally, it supplies sensory innervation to the anteromedial thigh. In males, it also contributes to the innervation of the anterior surface of the scrotum and root of the penis. The genitofemoral nerve (L1, L2) is commonly identified as it pierces the psoas major muscle and descends along its anterior surface. It then passes through the deep inguinal ring and into the inguinal canal, where it divides into respective genital and femoral branches superior to the inguinal ligament. The genital branch provides motor innervation of the cremaster muscle of the spermatic cord in males, while in females, this branch supplies the labia majora, adjacent thigh, and mons pubis. The femoral branch provides cutaneous innervation to the skin overlying the femoral triangle. The lateral femoral cutaneous nerve (LFCN) strictly provides sensory innervation to the skin of the lateral thigh, specifically between the hip and knee joints. This branch originates from the L2 and L3 spinal cord levels. It pierces through the psoas major muscle laterally as it traverses toward the inferior aspect of the anterior superior iliac spine, typically crossing superficial to the iliacus muscle and its associated fascia, before it then passes deep to the ilioinguinal ligament. The femoral nerve originates from the L2, L3, and L4 spinal cord levels, coursing laterally to the psoas major muscle and then inferiorly between the psoas major and iliacus. The femoral nerve supplies the iliacus and pectineus muscles in the pelvis, as well as providing motor and sensory innervation to the anterior compartment of the thigh. Lastly, the obturator nerve, which originates from the L2, L3, and L4 spinal cord levels, provides motor and sensory innervation to the medial compartment of the thigh. It resides in the pelvic space that is medial and deep to the psoas major muscle. While this organization of the lumbar plexus is the typical presentation, embryologic abnormalities can be a cause of the variations observed throughout populations.

Embryology of the lumbar plexus

Lumbar plexus development begins during weeks 4-8 of gestation, with the spinal cord and spinal nerves deriving from the neural tube [5]. During embryonic growth, the spinal nerves of the lumbar region elongate laterally and ventrally, intricately linked to the formation of the L1-L4 somites, which are portions of mesoderm that differentiate into skeletal muscle, vertebrae, and skin [5,8]. As somites differentiate, they guide the growth and segmentation of spinal nerves. The resulting somite-nerve interaction ensures that each somite segment receives proper innervation, aligning with the corresponding musculature and dermal regions [9]. After ventral migration of the growing nerves, the lumbar plexus will give rise to terminal branches that will innervate structures of the pelvis, thigh, and abdominal wall [5]. These branches include the femoral, obturator, iliohypogastric, ilioinguinal, and genitofemoral nerves, as well as LFCN. With further differentiation and maturation, these nerves contribute to the varying array of innervation described above [8].

Clinical presentations of lumbar plexus variations and injuries

The embryological development of the lumbar plexus influences the functional anatomy of the nerve branches that supply the lower limbs. As a result, it is imperative to understand how variation in this structure can impact the activities of daily living, surgical intra- and postoperative outcomes, or pain management. The lumbar plexus supports basic functions such as hip flexion, knee flexion and extension, and thigh adduction; this means that ADLs such as standing, sitting, or walking are directly impacted by the branches of the lumbar plexus, and in fact, the entire lumbar plexus and each branch derived from the lumbar plexus have been associated with clinical presentations of numbness or tingling along the lower extremity, muscle weakness, and difficulty completing the gait cycle due to illness, injury, or impingement [10-12]. For example, femoral neuropathy is characterized by patients presenting with pain and paresthesia in the anterior thigh, weakened thigh flexion, and impaired knee extension. Atrophy of the quadriceps may result from parturition, diabetic myelopathy, diseases spread from nearby joints or organs, or, most commonly, from compression or traction after abdominal, pelvic, or hip surgery [10,13]. Obturator neuropathy may also result from general surgeries and hip replacements, pelvic fractures, labor, or metastatic diseases, presenting with a loss of sensation and muscle weakness in the medial thigh [13,14]. LFCN neuralgia, or meralgia paresthetica, may result from entrapment of the LFCN near the inguinal ligament [15]. Patients typically present with numbness and pain in the anterolateral thigh. Variation in the spinal nerve contributions as well as the branching patterns and distribution of the LFCN results in variation in symptom variation and susceptibility to injury [16].

Understanding lumbar plexus anatomy and potential variations thereof is critical for preventing injury as well as treating injuries. Surgical trauma presents as one of the causes of lumbar plexus-associated neuropathies [17]. In managing surgical cases, it is crucial to be able to navigate nerve pathways in the lower abdominal cavity, the pelvic region, or during orthopedic procedures such as hip replacements to prevent unintended injury. Further, for many patients, treatment for lumbar plexus-associated neuropathies may include nerve release or blocks, the effectiveness of which is dependent on the clinician’s understanding of the developmental origins and the typical pathways of the lumbar plexus as well as the potential for supernumerary branches and variations in their distributions [18,19]. A knowledge of variation in the presentation and distribution of lumbar plexus-derived nerves, therefore, supports not only correct diagnosis but also ensures accurate and effective interventions while minimizing risk to the nerves.

## Case presentation

This case series included two human body donors identified in the gross anatomy laboratory at Midwestern University with unique anatomical variations in the branching patterns of the lumbar plexus. Both donors were initially dissected during gross anatomy courses and then dissected more intricately by our team, including both a superficial and deep dissection relative to the psoas major muscle. This study was determined not to meet the definition of human subjects research as defined in 45 CFR 46.102 by the Institutional Review Board of Midwestern University.

Case 1

**Figure 1:**
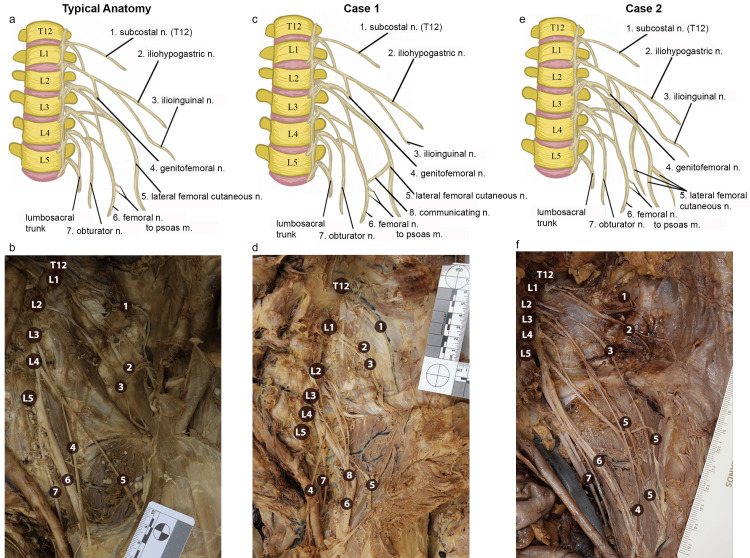
Left lumbar plexus arrangements: (a-b) Typical anatomical arrangement in line drawing illustration (a) and photograph of cadaveric donor (b). (c-d) Case 1 (83 M) in line drawing illustration (c) and photograph (d). (e-f) Case 2 (84 F) in line drawing illustration (e) and photograph (f) showing left lumbar plexus nerve branches. All images are oriented such that superior is at the top and lateral is to the right. Numbers indicate: (1) subcostal nerve, (2) iliohypogastric nerve, (3) ilioinguinal nerve, (4) genitofemoral nerve, (5) LFCN, (6) femoral nerve, (7) obturator nerve, (8) communicating nerve between the lateral femoral cutaneous and femoral nerves. T12, L1, L2, L3, L4, and L5 indicate anterior rami and their associated vertebral body. (a-c) Image Credit: Authors, created using Adobe Photoshop (Adobe Inc., San Jose, CA, USA) and Fresco (Thermo Fisher Scientific, Waltham, MA, USA) LFCN: lateral femoral cutaneous nerve

LFCN received contributions only from the L2 spinal nerve, with a communicating branch to the femoral nerve. In the first case, an 83-year-old male presented with an atypical arrangement in the branching pattern of the anterior rami forming LFCN. The LFCN was composed exclusively of two separate branches from L2, forming as the L2 anterior ramus bifurcated into the femoral nerve and LFCN (Figure 1c-1d). L3 and L4 anterior rami both contributed to the femoral nerve as expected, which in turn provided additional nerve communications with the LFCN. Additionally, the ilioinguinal nerve was noticeably smaller than the iliohypogastric nerve (Figure 1c-1d).

Case 2

LFCN receives contributions from the L1 to L3 spinal nerves and may have supernumerary branches. In the second case, an 84-year-old female (Figure 1e-1f), the LFCN received a contribution from L1, in addition to the typical L2-L3 contributions, which traveled inferiorly before joining at the level of L5. The L1 contributions descended inferiorly, parallel to the genitofemoral nerve, before joining with another branch of LFCN that originated from L3 and diving deep into the ilioinguinal ligament. These branches subsequently separated into distinct branches and ultimately innervated discrete areas in the thigh. The femoral nerve was also composed of multiple individual nerve branches of L2-L4 coursing together inferiorly and lateral to the psoas major muscle, which branched off more proximally than usual in the pelvis. In addition, a thin branch of the femoral nerve coursed inferiorly parallel to the obturator nerve and medial to the psoas major muscle.

## Discussion

Variations documented here provide examples of the complex nature of the lumbar plexus and the need to document additional cases of variation. Of particular note was the variation in LFCN, ranging from variations in spinal cord level contributions to the number of branches to the patterns of distribution. A recent study discovered atypical spinal nerve origins of the LFCN in 14.7% of lumbar plexuses, with 2.9% receiving contributions solely from L2 [20]. A further meta-analysis discovered that LFCN spinal nerve contributions ranged from L2-L3 in 71.6%, L2 only in 10.2%, L2-L4 in 2.9% L1-L3 in 1%, L3 in 1%, L1 in 0.7% and T12-L1 in 0.2% of the donors and patients studied, suggesting that variation in the LFCN is not a unique phenomenon among our donors [4]. The implications of unexpected anatomical variation of the lumbar plexus are significant due to the potential for nerve entrapment and neuropathic pain, as well as its role in nerve block administration and surgical procedures [21,22]. In particular, unexpected supernumerary LFCN branches may lead to increased risk of entrapment and/or surgical complications.

Compression of the LFCN in its standard distribution is relatively common [12,20], often resulting in meralgia paresthetica, a condition associated with pain, aching, and burning sensations of the anterolateral thigh [23,24]. Symptoms may be the result of compression/entrapment from a hypertonic psoas major, inguinal ligament, tight clothing, heavy tool belts, weight gain, seatbelt injuries from car accidents, or even metabolic disorders, such as diabetes and hypothyroidism [12,23,25-27]. In the case of a variable LFCN like those seen here, some of its branches may be located superficial to the inguinal ligament, resulting in even greater susceptibility to damage/injury. For cases of chronic meralgia paresthetica without relief from conservative treatments, surgical decompression, in which the LFCN is located and freed from the surrounding tissue, may alleviate symptoms [28]. The multitude of LFCN branches observed here suggests that this procedure may be ineffective if only a single nerve branch is targeted; rather, it may be necessary to locate and decompress supernumerary LFCN branches to ensure adequate symptom relief.

Arthroscopic surgeries of the hip are the most common cause of injury to the LFCN, including paresis or anesthesia due to laceration of the nerve [29]. In a recent retrospective study of 179 patients who underwent hip arthroplasty, 77 (43%) experienced LFCN injury, including 92% of patients with a repeated procedure [29]. In hip arthroscopy, there are key points at which the surgical team must be especially careful of the LFCN, including the incision of fascia superficial to the tensor fascia latae parallel to its fibers toward the sartorial/tensor fascia latae plane and when suturing this fascia during closure [29]. Patients with LFCN variations, such as those described above, may be at higher risk of iatrogenic damage during these steps due to the unexpected location and number of LFCN branches.

A diagnostic tool to identify the location of and possible variations in the LFCN is through the use of ultrasonography to determine its 3D distribution. Ultrasound or other imaging can significantly reduce sensory deficits and injury to the LFCN during surgery or a nerve block procedure [8,30]. This tool is commonly used in medical procedures to assess variation in LFCN and target its branches accurately [31-34]. The variations observed here further support the need to have a full understanding of the distributions and branching patterns of the LFCN and surrounding structures in patients prior to clinical procedures.

## Conclusions

The anatomical variation found here within the lumbar plexus, particularly the variation in spinal nerve contributions and branching patterns of the LFCN, adds to a growing body of literature describing such anatomical variation across human donors and patients. Variation within the LFCN specifically may result in an increased likelihood of compression of the nerve due to branches passing near the psoas major or superficial to the inguinal ligament. Supernumerary branches of the LFCN may also add challenges to nerve blocks and nerve decompression, resulting in difficulty in providing relief in cases of chronic meralgia paresthetica. Variation in LFCN distribution may also increase its likelihood of damage in hip surgeries. The variations in spinal nerve contributions and branching patterns found in the lumbar plexus of these two donors add to a growing body of literature that provides insight for clinicians and, therefore, contributes to improved patient outcomes and care.
